# Personalized Automation of Treatment Planning for Linac-Based Stereotactic Body Radiotherapy of Spine Cancer

**DOI:** 10.3389/fonc.2022.824532

**Published:** 2022-02-02

**Authors:** Savino Cilla, Francesco Cellini, Carmela Romano, Gabriella Macchia, Donato Pezzulla, Pietro Viola, Milly Buwenge, Luca Indovina, Vincenzo Valentini, Alessio G. Morganti, Francesco Deodato

**Affiliations:** ^1^ Medical Physics Unit, Gemelli Molise Hospital - Università Cattolica del Sacro Cuore, Campobasso, Italy; ^2^ Radiation Oncology Department, Fondazione Policlinico Universitario A. Gemelli - Università Cattolica del Sacro Cuore, Roma, Italy; ^3^ Radiation Oncology Unit, Gemelli Molise Hospital - Università Cattolica del Sacro Cuore, Campobasso, Italy; ^4^ Radiation Oncology, Istituti di Ricovero e Cura a Carattere Scientifico (IRCCS) Azienda Ospedaliero-Universitaria di Bologna, Dipartimento di Medicina Specialistica, Diagnostica e Sperimentale (DIMES), Alma Mater Studiorum Università di Bologna, Bologna, Italy; ^5^ Medical Physics Unit, Fondazione Policlinico Universitario A. Gemelli - Università Cattolica del Sacro Cuore, Roma, Italy; ^6^ Istituto di Radiologia, Università Cattolica del Sacro Cuore, Roma, Italy

**Keywords:** automated planning, spine, stereotactic body radiation therapy (SBRT), volumetric modulated arc therapy (VMAT), pinnacle

## Abstract

**Purpose/Objective(s):**

Stereotactic ablative body radiotherapy (SBRT) for vertebral metastases is a challenging treatment process. Planning automation has recently reported the potential to improve plan quality and increase planning efficiency. We performed a dosimetric evaluation of the new Personalized engine implemented in Pinnacle3 for full planning automation of SBRT spine treatments in terms of plan quality, treatment efficiency, and delivery accuracy.

**Materials/Methods:**

The Pinnacle3 treatment planning system was used to reoptimize six patients with spinal metastases, employing two separate automated engines. These two automated engines, the existing Autoplanning and the new Personalized, are both template-based algorithms that employ a wishlist to construct planning goals and an iterative technique to replicate the planning procedure performed by skilled planners. The boost tumor volume (BTV) was defined as the macroscopically visible lesion on RM examination, and the planning target volume (PTV) corresponds with the entire vertebra. Dose was prescribed according to simultaneous integrated boost strategy with BTV and PTV irradiated simultaneously over 3 fractions with a dose of 30 and 21 Gy, respectively. Dose-volume histogram (DVH) metrics and conformance indices were used to compare clinically accepted manual plans (MP) with automated plans developed using both Autoplanning (AP) and Personalized engines (Pers). All plans were evaluated for planning efficiency and dose delivery accuracy.

**Results:**

For similar spinal cord sparing, automated plans reported a significant improvement of target coverage and dose conformity. On average, Pers plans increased near-minimal dose D98% by 10.4% and 8.9% and target coverage D95% by 8.0% and by 4.6% for BTV and PTV, respectively. Automated plans provided significantly superior dose conformity and dose contrast by 37%–47% and by 4.6%–5.7% compared with manual plans. Overall planning times were dramatically reduced to about 15 and 23 min for Pers and AP plans, respectively. The average beam-on times were found to be within 3 min for all plans. Despite the increased complexity, all plans passed the 2%/2 mm γ-analysis for dose verification.

**Conclusion:**

Automated planning for spine SBRT through the new Pinnacle3 Personalized engine provided an overall increase of plan quality in terms of dose conformity and a major increase in efficiency. In this complex anatomical site, Personalized strongly reduce the tradeoff between optimal accurate dosimetry and planning time.

## Introduction

The spinal vertebrae are a common location for metastases from many primary cancers (1), leading to pain and neurologic dysfunction. Conventional fractionated radiation therapy has been the standard treatment of the palliative management, but long-term control of symptoms has been reported to be at best approximately 60%, with a median duration of palliation of 4 months (2).

The ineffectiveness of traditional radiation is mostly due to technological limitations, which limit the treatment dose to values below those required for tumor ablation. In the last years, the technological advances in treatment dose delivery with intensity-modulated techniques (IMRT), image-guided systems (IGRT), and new treatment planning optimization algorithms entailed new abilities to achieve a high precision of target coverage with tumoricidal dose levels, while sparing the spinal cord. These technological advancements facilitated the application of stereotactic ablative body radiation therapy (SBRT) for vertebral metastasis (3). Phase I–II studies demonstrated clinical benefits of SBRT in the primary or salvage treatment of stable spinal lesions (4). The results of the RTOG 0631 study showed SBRT to be feasible and accurate (5). In particular, it has been reported that the risk of radiation myelopathy can be kept to ≤1% with high-technological planning and delivery systems (6). Furthermore, radiation dose was found to be a strong predictor of local control in trials on dose escalation (7); spine SBRT has resulted in 80%–90% local control rates after 1 year in many published *de novo* and adjuvant studies (8, 9).

Despite technological advancements, concern have been raised about the most effective dose-fractionation schemes in spine SBRT because, when compared with conventional radiotherapy, the risk of vertebral compression fractures (VCF) has been shown to be as high as 40% (10). The ideal dose fractionation for spine SBRT is at present uncertain. Different schedules are common in clinical practice including 18 to 24 Gy in 1 fraction, 24 Gy in 2 fractions, 24 to 30 Gy in 3 fractions, 30 Gy in 4 fractions, and 30 to 40 Gy in 5 fractions. Nowadays, no randomized trials confirmed the superiority of single-fraction SBRT as compared with multiple-fraction SBRT. A simultaneous integrated boost (SIB) dose delivery approach has been proposed to reduce the risk of SBRT-induced VCF by increasing the gross tumor volume and including nonaffected bone in the clinical target volume. This strategy has not yet been fully explored and only very few papers demonstrated that SIB strategy could be successfully applied to spinal metastases in a dose-escalation trial (11–13).

SBRT planning optimization for spinal metastases remains a high challenging task because of the complex relationship between the vertebral segment and the adjacent critical structures, particularly the spinal cord. Treatment planning for spine SBRT necessitates extensive skill and a significant time investment on the part of treatment planners. Three recent multicentric studies investigated the dosimetric variability in SBRT planning across a large number of centers. Esposito et al. (14) reported the dosimetric variability in spine SBRT planning in a large number of centers in order to identify crowd knowledge-based solutions. With comparable planning/delivery technologies, planners produced significant different plans in terms of quality, highlighting the importance of the skills of planners in the SBRT planning optimization process and the utility of knowledge sharing to increase plan quality. Moustakis et al. (15) reported the results of a comparative planning study for vertebral SBRT using different treatment platforms emphasizing that target and critical structural dosimetry discrepancies were substantially planner dependent rather than system dependent. Hardcastle et al. (16) reported the findings of an international planning challenge on a vertebral SBRT case, illustrating once more that high-quality were dependent on planner skills rather than technologically advanced in planning systems.

Artificial intelligence applications in radiation oncology are already translating into rapid technological breakthroughs in several elements of the radiotherapy process, including patient outcome modeling, organ autosegmentation, dose prediction, and treatment plan automation (17, 18). However, until today, only few studies intended to validate full automated VMAT plan engines for spinal metastases. Buergy et al. (19) evaluated the performance of the Erasmus-iCycle algorithm to automatically generate high-quality VMAT plans for SRS/SBRT treatments of spinal metastases. At the cost of somewhat longer treatment periods, automated plans provided higher target coverage and OAR sparing. Foy et al. (20) explored the feasibility of knowledge-based planning strategy using Varian RapidPlan software for 10 spine SBRT cases, reporting an overall improved quality and a decrease of total planning time from approximately 1.5 h to 15 min. Last, a dedicated TPS, the Elements Spine SRS (Brainlab AG, Munich, Germany), has been recently evaluated for the generation of automated quality plans in spine SBRT (21, 22). Comparison with previous clinical plans obtained with other TPS resulted in sharpest dose gradient and in lower spinal cord maximum point doses.

A commercial solution, the Autoplanning module implemented in the Pinnacle3 treatment planning system (Philips Medical Systems, Fitchburg, WI, USA), has been successfully used to automate the treatment planning process and find the best patient treatment plan. This strategy is based on a template-based planning optimization procedure that employs an iterative approach capable of replicating all processes of experienced and skilled planners in order to generated high-quality plans (23). The Autoplanning module has been recently evaluated for SBRT treatment of liver and pancreatic tumors, showing a major ability to deliver ablative doses respecting all normal tissue constraints with respect to manually generated plans (24, 25).

Pinnacle3 Personalized, a new generation of advanced algorithms, is currently being investigated with the goal of improving overall plan quality and the speed of IMRT and VMAT optimization for automated plan generation. In particular, the Personalized engine integrates an advanced technology called Feasibility that allows the estimation of the best feasible organ-at-risk dose sparing, then providing an “*a priori*” knowledge about the achievability of treatment planning goals. The first clinical application of this new strategy has been recently tested for challenging treatments of head-neck and prostate cancers, reporting a general improvement in plan quality in terms of dose conformity and normal tissue sparing (26, 27).

Based on the recent literature data, we aimed to investigate the feasibility of the new Pinnacle3 Personalized engine in creating challenging treatment plans for spine SBRT using a SIB approach and to test the hypothesis that high-quality automated generated plans can be created more efficiently than manual plans. This scenario represents an extreme technical treatment planning challenge, due to the irregular-shaped target volume, the location of critical OARs immediately adjacent to the target structures, and the delivery of heterogeneous dose distribution.

In particular, following the PREST trial (NCT03597984) protocol (28), in this study, we hypothesize the feasibility of SBRT automated planning to administer, in three treatment fractions, 21 and 30 Gy to the whole vertebra and to the macroscopic lesion, respectively. This SIB strategy should then allow radiation dose escalation without increasing the risk of radiation-induced myelopathy and VCF.

## Material and Methods

### Patient Population, Volume Definition, and Dose Prescription

Six patients were retrospectively selected from our institutional review board. Patient data were anonymized and de-identified to protect patient confidentiality. Patients presented varied tumor sites to represent several complex clinical settings for SBRT planning. **Table 1** lists the details of the locations and volumes of the lesions.

**Table 1:** Summary of the locations and BTV and PTV volumes of the lesions.

Patients	Site	BTV (cc)	PTV (cc)
1	L1	11.8	92.6
2	L2	19.8	106.3
3	L2	5.1	103.9
4	D11	8.4	80.8
5	L1	3.0	81.0
6	C4	1.6	19.8

All patients had a simulation computed tomography (CT) scan (2-mm slice thickness) in the supine position at 3-mm interval from the vertex to the level of the aortic arc.

**Figure 1:**
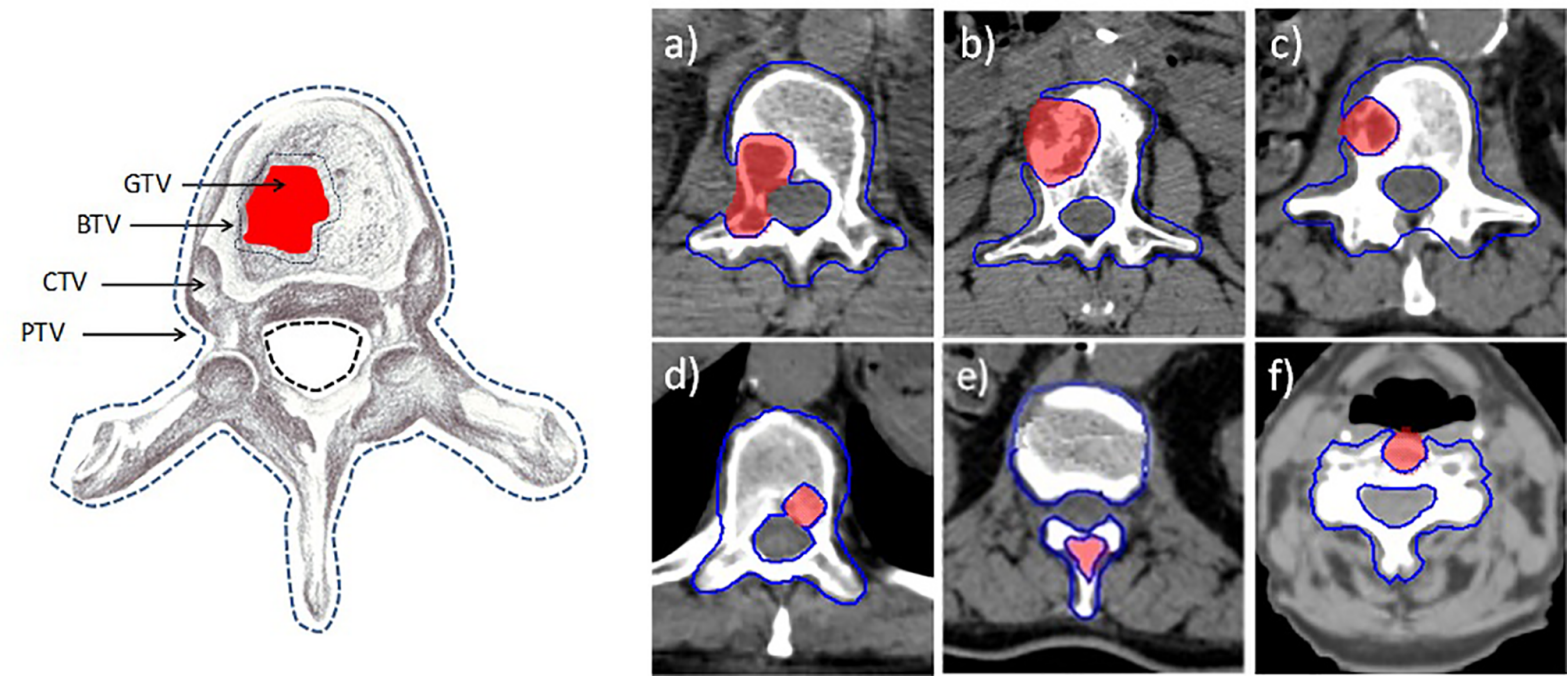
Definition of target volumes. GTV, gross target volume; BTV, boost target volume; CTV, clinical target volume; PTV, planning target volume. On the left, the six axial images **(A–F)** refer to the six patient lesions reported in **Table 1**.

A PET-CT and MRI imaging was coregistered with the simulation CT for accurate volume delineation. The gross tumor volume (GTV) was defined as the macroscopically visible lesion on MRI examination. The clinical target volume (CTV) corresponds with the entire vertebra. The boost target volume (BTV) and the planning target volume (PTV) were defined by expanding the GTV and the CTV by 2 mm, respectively. Two planning organ-at-risk volumes for spinal cord were generated as 1- and 2-mm uniform expansions and were denoted cord_1mm and cord_2mm. These margins were employed to account for both image-guided spinal SBRT setup uncertainty and spinal cord motion or position within the thecal sac (29). The pictorial definition of target volumes is shown in **Figure 1**.

Radiotherapy was prescribed according to SIB strategy with BTV and PTV irradiated simultaneously over 3 fractions. Doses of 30 Gy (10 Gy/fraction) and 21 Gy (7 Gy/fraction) were prescribed to the BTV and PTV, respectively. This dose fractionation was based on the PREST phase III randomized multicentric trial (28) recommendations. The optimal coverage for BTV and PTV was considered: D95% ≥95% of each prescription dose. Although dose inhomogeneity in BTV and PTV was not considered a priority, a particular attention was paid in order to obtain any dose ≥105% of prescription dose within the target volumes.

Compliance of spinal cord to the maximum dose was considered optimal if D_0.03cc ≤_18.8 Gy, where D_0.03cc_ is the dose to the 0.03 cc volume and represents the near-maximal dose. Using this constrain and under the assumption of the linear quadratic model and an *α*/*β*-value of 2 for late effect, the probability of myelopathy was kept below 3% (30). This constraint is more restrictive than the one suggested by the AAPM101 (31) guidelines, that is D_0.03cc ≤_21.9 Gy, which was instead used as a hard constraint, together with the additional constraint D_0.35cc ≤_18.0 Gy (31). All three constraints were simultaneously used in the planning optimization process. Maximal doses for other OARs (esophagus, heart, lungs, trachea, liver) were to be kept within the suggested guidelines of AAPM101.

### Treatment Planning

Three plans were created for each patient using the VMAT optimization technique for coplanar flattening filter-free (FFF) 6MV photon beams from an Elekta VersaHD linac (Elekta Ltd., Crawley, UK), equipped with the Agility 160-leaf multileaf collimator. An expert medical physicist created the manual VMAT plans (MP plans) according to local protocols. Pinnacle3 Autoplanning and Pinnacle3 Personalized modules were used to create automated VMAT plans, which were compared with the clinically accepted ones. The dose calculations were done with a 2-mm grid resolution using the collapsed cone convolution dose calculation algorithm. A “dual-arc” configuration was used to generate all plans; a full gantry rotation was represented by a sequence of 180 control points, one every 2°.

### Pinnacle3 Autoplanning

The Autoplanning module in Pinnacle3 TPS version 16.2 was used to automatically generate the AP plans for each patient, utilizing a template based on the identical beam parameters, dose prescription, and clinical objectives as manual plans. This module was previously described in details (20). Briefly, this module employs a “Technique,” which is a set of adjustable parameters, including the definition of beam characteristics, dose prescriptions, and planning objectives for PTVs and OARs, that may be tailored to each treatment protocol and tumor site. The Technique is used by the Autoplanning engine to iteratively optimize planning parameters in order to best fulfill the required planning goals. Several dummy structures are automatically generated by the Autoplanning engine during the optimization process including (a) rings around the PTVs to manage the dose fall-off, (b) residual targets structures where overlaps between no-compromised OARs are removed, (c) residual OARs structures where overlaps between targets are removed, (d) body structures used to control body dose, and (e) hot-spot and cold-spot structures to manage target dose uniformity.

An iterative process is then carried out over multiple optimization loops by the Autoplanning engine that automatically adds new objectives to these structures and adjusts the optimization parameters in order to continuously spare the OARs without compromising the target coverage, then simulating what a manual experienced planner would normally do.

### Pinnacle3 Personalized

New automated plans (Pers_plan) were created using a new generation of automated treatment planning technology called Personalized and implemented in the Pinnacle3 Evolution TPS version 16.4. The Pinnacle3 Personalized module was previously described in greater details (26). Briefly, this module combines new powerful Philips-exclusive optimization algorithms with the so-called Feasibility technology. Two new powerful algorithms are now available in order to enhance the efficiency of automation process, namely the Broyden-Fletcher-Goldfarb-Shanno (L-BFGS) algorithm for fluence map optimization and the Layered Graph algorithm used for aperture size and shape optimization. One of the main features is the integration of a new module called Feasibility within the optimization process, able to provide the best-case scenario dose distribution for any patient. This module creates a “feasibility” dose-volume histogram (fDVH) for each OAR based on the patient’s CT imaging, prescription doses, and the geometric relationship between the target volumes and OAR. The planner is then given the *a priori* optimal dose distribution and the achievability of treatment planning goals (26). The Personalized engine then integrates the Feasibility goals into the Technique to iteratively tune planning parameters to best fulfill the specified planning goals.

For both AP and Pers plans, after the end of automated optimization, a manual fine-tuning of the plans was performed if hot-spots, i.e., local small-dose areas exceeding dose objectives, were still present within Cord_2mm.

### Plan Evaluation

Dose-volume histograms were used to compare all of the plans. The target volumes coverage was compared in terms of mean doses, D98% (the near-minimal dose), D95%, D50% (the median dose), and D2% (the near-maximal dose). OAR dose sparing was evaluated following the metrics previously reported.

The dose conformity indexes (CI95%) to each target volume were calculated as the ratio of the tissue volume covered by the reference isodose and the volume of that target. For the ideal case, CI = 1. We also calculated the dose conformity at 50% for the vertebra (CI50%) as the ratio between the tissue volume receiving 50% of the prescription doses and the PTV volume, in order to obtain a volumetric measure of how rapidly the dose falls off from the prescription isodose line.

The dose contrast index (DCI) was utilized to quantify the ability to deliver highly heterogeneous doses to the two target volumes as requested for the SIB method (32). The ideal DCI (iDCI) was calculated as the ratio of prescription doses to the BTV and the PTV and was then equal to 1.429. The DCI was calculated by dividing the mean dose to the BTV by the mean dose to the PTV. The percentage DCI (percent DCI) is defined as the ratio of DCI and iDCI multiplied by 100 and quantifies the deviation of the real DCI from the ideal iDCI. A dose contrast closer to 100% suggests a superior contrast ability.

### Planning Efficiency and Plan Complexity

The cost effectiveness of the planning procedure was assessed for each patient by examining total planning time (human inputs, optimization loops, and dose calculation times), treatment delivery time, and total number of monitor units. All optimization processes were carried out on a centralized server architecture (Oracle Pinnacle Professional X6-2, 22-core 2.20 GHz processor).

### Dose Delivery Verification

The accuracy of dose deliverability was assessed by a dosimetric verification of all plans. The 1000SRS ion-chamber array and the Octavius-4D phantom, both developed PTW (PTW, Freiburg), were used to measure dose distributions. The 1000SRS array consists of 977 liquid-filled ion chambers organized in a grid over a 11 × 11 cm^2^ area. This array is inserted into the Octavius-4D motorized cylindrical polystyrene phantom, which can rotate synchronously with the gantry enabling 3D dose reconstruction. The gamma-index metric was used to compare the measured and calculated dose distributions. Dosimetric verification was judged optimal if the percentage of points fulfilling gamma index criteria exceeded 90%, using a global dose criterion of 3% and a distance-to-agreement threshold of 2 mm, according to the recent recommendations of the AAPM study No.218 (33).

### Statistical Analysis

A Kruskal-Wallis analysis of variance (ANOVA) was used to compare the data, with adjusted *p*-values of 0.05 indicating statistical significance.

## Results

### Target Coverage

**Tables 2** and **3** report the dosimetric data for the BTV and PTV coverage and dose conformity. Automated Pers plans resulted in a statistically significantly improvement of near-minimal dose (D98%) and target coverage (D95%) for both target volumes (*p* < 0.05). In particular, Pers plans increased near-minimal dose D98% by 10.4% and 8.9% and target coverage D95% by 8.0% and by 4.6% for BTV and PTV, respectively. AP and Pers plans significantly improved dose conformity when compared with MP plans, suggesting a higher potential to better conform doses to this complex anatomy. In particular, automated plans provide significantly superior dose conformity and dose contrast by 37%–47% and by 4.6%–5.7% compared with manual plans. No significant differences in all dosimetric metrics were found between AP and Pers plans.

**Table 2:** Summary of dosimetric data for the BTV and PTV coverage.

	MP	AP	Pers	*p*	*p*		
				Kruskal-Wallis	MP vs. AP	MP vs. Pers	AP vs. Pers
BTV							
D98%	26.0 ± 3.0	28.5 ± 2.0	28.7 ± 2.1	0.080	0.061	**0.045**	0.897
D95%	27.5 ± 2.1	29.1 ± 1.5	29.7 ± 1.3	0.056	0.111	**0.019**	0.451
D50%	29.9 ± 1.1	31.2 ± 0.2	31.2 ± 0.5	0.774	0.516	0.948	0.559
D2%	32.9 ± 1.3	32.1 ± 0.7	31.8 ± 0.7	0.088	0.143	**0.031**	0.490
PTV							
D98%	18.0 ± 2.2	19.1 ± 1.7	19.6 ± 1.6	0.140	0.291	**0.047**	0.354
D95%	19.6 ± 1.3	20.3 ± 1.2	20.5 ± 1.2	0.171	0.203	0.067	0.575
D50%	23.1 ± 0.3	22.5 ± 0.2	22.1 ± 0.5	**0.001**	**0.021**	**<0.001**	0.161
D2%	30.4 ± 0.8	29.8 ± 0.8	29.9 ± 0.6	**0.042**	**0.023**	**0.038**	0.846

Bold values are statistically significant values.

**Table 3:** Summary of dosimetric data for dose conformity and contrast.

	MP	AP	Pers	*p*	*p*		
				Kruskal-Wallis	MP vs. AP	MP vs. Pers	AP vs. Pers
CI95 BTV	2.8 ± 1.0	2.1 ± 0.6	1.9 ± 0.6	0.064	0.131	**0.021**	0.425
CI 95 PTV	2.6 ± 0.8	2.1 ± 0.5	1.9 ± 0.5	**0.033**	0.089	**0.010**	0.389
CI50 PTV	8.3 ± 1.2	7.3 ± 0.9	6.6 ± 0.9	**0.017**	**0.041**	**0.006**	0.491
DCI	92.5 ± 1.9	97.1 ± 0.7	98.2 ± 1.3	**0.001**	**0.010**	**<0.001**	0.227

Bold values are statistically significant values.

**Figure 2:**
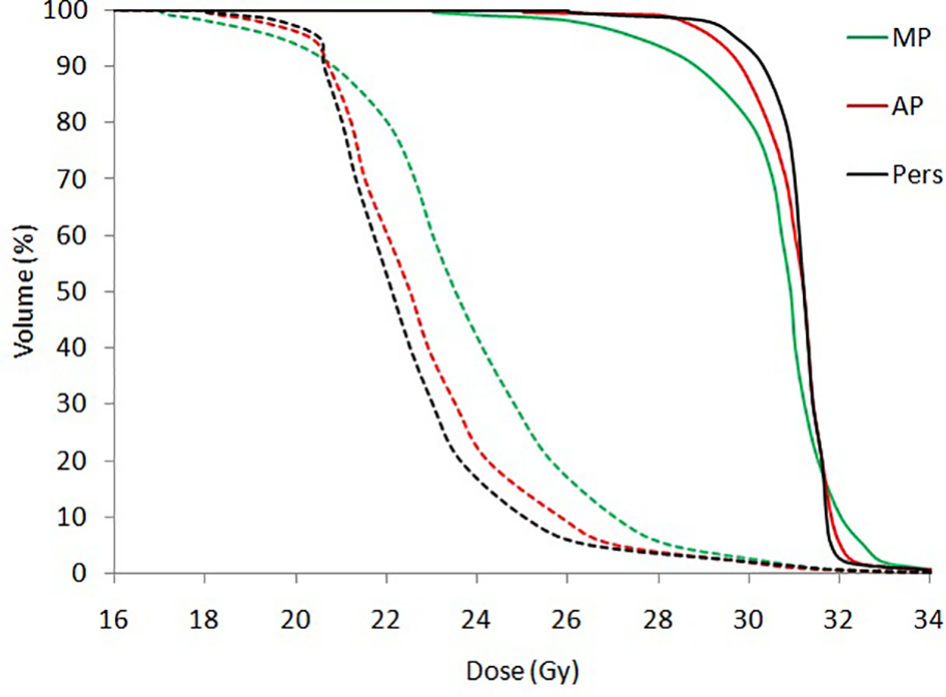
Mean dose volume histograms (DVHs) for BTV (solid lines) and PTV (dashed lines) for all patients.

**Figure 2** shows the average dose-volume histograms (DVHs) for all patients for BTV (solid lines) and PTV (dashed lines).

**Figure 3:**
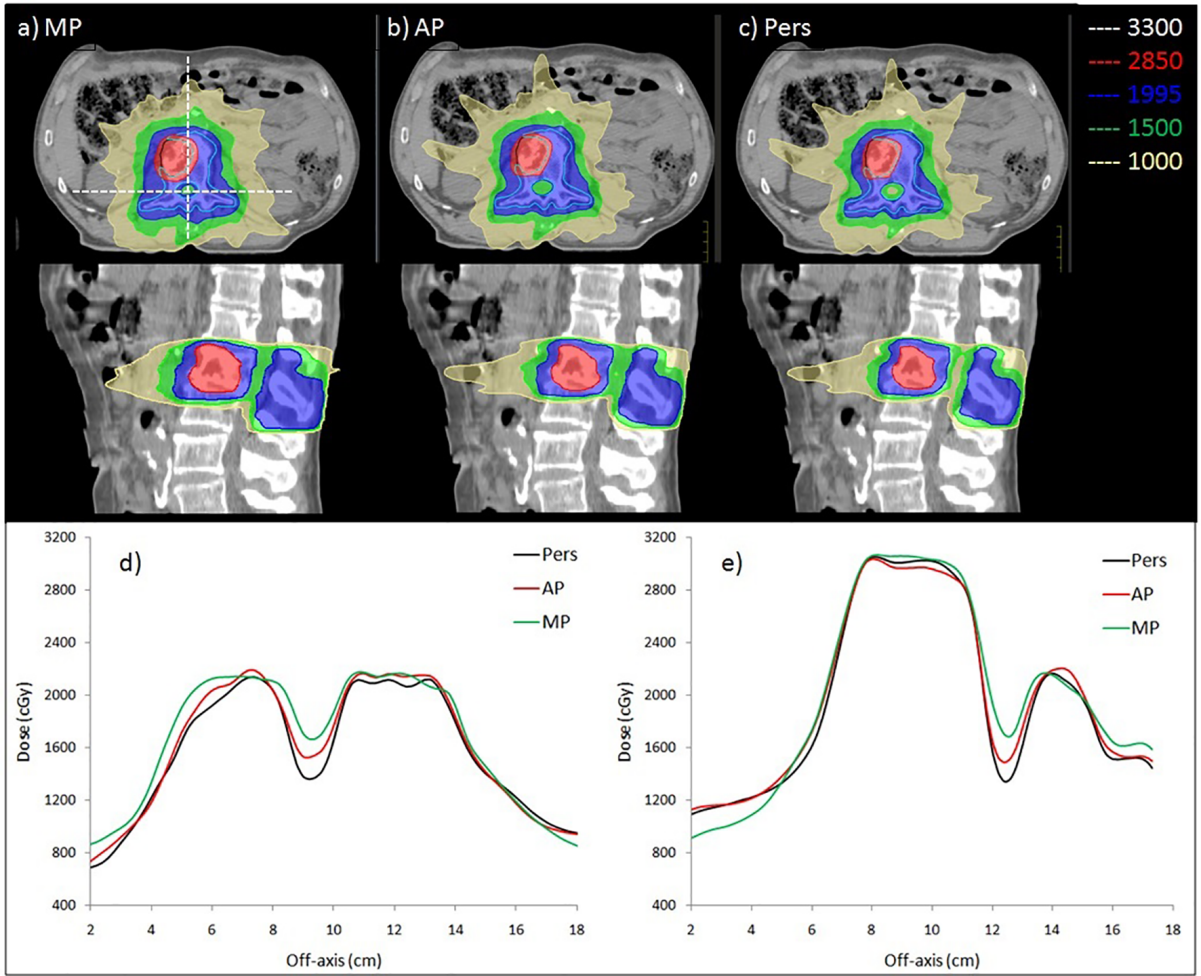
Comparison of dose distribution obtained by **(A)** manual, **(B)** Autoplanning and **(C)** Personalized plans in axial and sagittal planes for case 2. The BTV and PTV are delineated by black and light blue contour. Isodose curves are shown for 3,300 cGy (white, 110% of BTV prescription), 2,850 cGy (red, 95% of BTV prescription), 1,995 cGy (blue, 95% of PTV prescription), 1,500 cGy (green, 50% of BTV prescription), and 1,000 cGy (yellow, low-dose bath). The bottom panel shows the 2D dose profile along **(D)** horizontal and **(E)** vertical lines crossing through the cord, as shown in **(A)**.

**Figure 3** shows the isodose distributions for (a) MP, (b) AP, and (c) Pers plans for representative patients (case 2) in axial and sagittal planes. In **Figure 3** **(**e and d), the horizontal (in left-right direction) and the vertical (in anterior-posterior direction) dose profiles along the dashed line drawn on the axial plan are reported to highlight the differences of dose gradient for MP, AP, and Pers plan, respectively.

### OAR Sparing

**Table 4** reports the dosimetric data for the spinal cord and skin sparing. As expected, since spinal cord maximal dose was forced as a major hard constraint during plan optimization, no significant difference were observed among MP, AP, and Pers plans. For the cord_2mm, the AAPM101 maximal dose objective of 21.9 Gy was satisfied in all plans. The maximum dose objective of 18.8 Gy for the spinal cord was met in four patients for the AP and Pers plans, but only one patient for the MP plans. For skin, Ap and Pers plans yielded a near-maximal dose lower by 8% (1.6 Gy) with respect to MP plans. All other organs-at-risk (heart, lungs, esophagus, etc.) involved for a given patient in the treatment field was well below the recommended dose tolerances.

**Table 4:** Summary of dosimetric data for spinal cord and skin dose sparing.

Near-maximal doses	MP	AP	Pers	*p*	*p*		
				Kruskal-Wallis	MP vs. AP	MP vs. Pers	AP vs. Pers
D_0.03_ (Gy)							
Cord	19.3 ± 1.6	18.2 ± 1.7	18.6 ± 1.1	0.348	0.167	0.281	0.763
Cord_1mm	21.1 ± 0.9	20.1 ± 1.3	20.4 ± 0.9	0.110	0.047	0.115	0.681
Cord_2mm	21.8 ± 0.3	21.4 ± 0.4	21.5 ± 0.3	0.090	0.047	0.103	0.648
D_0.35_ (Gy)							
Cord	15.9 ± 1.2	15.0 ± 1.6	15.4 ± 1.6	0.482	0.233	0.664	0.448
Cord_1mm	16.8 ± 0.6	16.5 ± 0.9	16.5 ± 0.9	0.872	0.626	0.684	0.935
Cord_2mm	17.3 ± 0.4	16.9 ± 0.4	17.1 ± 0.3	0.104	0.054	0.082	0.849
Skin	19.9 ± 0.9	18.3 ± 1.7	18.3 ± 1.7	**0.047**	**0.041**	**0.026**	0.863

Bold values are statistically significant values.

### Planning Efficiency and Dosimetric Verification

**Table 5** shows a detailed analysis of planning and treatment efficiency and quality of dosimetric verification. Data are presented as the mean and standard deviations for all patients. For Pers plans, the mean total number of MUs per fraction was found higher by 303 MUs (9.3%) and 669 MUs (23.0%) compared with AP and MP plans, respectively (*p* < 0.05 in both comparisons). Despite the large differences in MUs, the increase in MUs translated into longer treatment times by only 0.6 and 0.3 min with respect to AP and MP treatment times (*p* < 0.05 in both comparisons). Anyway, no treatment was longer than 3.0 min for a single treatment fraction.

**Table 5:** Summary of results for planning and treatment efficiency and delivery accuracy.

	MP	AP	Pers	*p*	*p*		
				Kruskal-Wallis	MP vs. AP	MP vs. Pers	AP vs. Pers
MUs	2,908 ± 278	3,273 ± 175	3,577 ± 292	**0.001**	**0.035**	**<0.001**	0.111
Treatment time (min)	2.4 ± 0.2	2.7 ± 0.1	3.0 ± 0.2	**0.001**	**0.022**	**<0.001**	0.132
Planning time (min)	161.5 ± 23.4	23.4 ± 1.4	15.6 ± 0.7	**<0.001**	**<0.001**	**<0.001**	**0.035**
GPR (2 mm–3%)	96.3 ± 1.6	94.7 ± 0.9	93.9 ± 1.4	**0.006**	**0.040**	**0.002**	0.281

Bold values are statistically significant values.

When switching from manual to automated planning, the average planning time was shown to decrease dramatically. AP and Pers plans were generated in roughly 23 and 15 min, respectively, using a centralized server architecture.

All plans underwent pretreatment verification. The average passrate for all plans and all planning methodologies was greater than 90% with criteria equal to 3% (global)/2 mm.

## Discussion

Spine SRS/SBRT planning is extremely challenging, and advanced treatment planning solutions are requested to generate the complex dose distributions needed in this clinical setting. The need to spare the spinal cord irradiation requires very steep dose gradients and high concave shape doses in order to avoid any overdosage to the spinal cord. The resulting treatment plans usually presented a very high degree of modulation, then pushing the limits of the accuracy of the treatment planning system and delivery platform. A few multicentric studies then focused on various planning strategies and delivery platform to improve quality in spine SRS/SBRT (14, 15). These studies well reported that all modern treatment planning systems and treatment techniques are able to perform spine SRS/SBRT treatments, but “it is rather the users experience and understanding of the optimization system that appears to be the driving factor for plan quality.”

These findings result in a significant plan variability because the choice of dosimetric tradeoffs in manual planning is planner dependent, and a long sequence of compromises must be individually negotiated for each patient in a trial-and-error process. Because the dose distribution that maximizes the therapeutic ratio for a given patient is never known *a priori*, not only do the resulting manual plans take a long time to develop, but they often result in sub-optimal plans, which can lead to poor patient outcomes (34). This issue motivated a need to increase spine SRS/SBRT planning efficiency and standardization, regardless of planner expertise.

To our knowledge, no researches investigated the use of template-based automated technique for spine SBRT treatments. In particular, we examined for the first time the potential of the new fully automated engines for VMAT planning deployed in the Pinnacle3 Personalized TPS for spine SBRT. The results of the present analysis provided new data in support of automation for challenging clinical scenarios as spine SBRT. We demonstrated the effectiveness of template-based planning for this anatomical site, able to generate plans with improved quality compared with those created by experienced medical physicist in only a fraction of the time. Plan quality was significantly for automated plans, particularly in terms of dose conformity to target volumes, with Pers plans outperforming AP plans. In particular, the use of the new Personalized engine with its Feasibility module allows an *a priori* knowledge of the theoretical dose-volume space available for the critical structures, able to identify for each different anatomical configuration the dosimetric outliers and planning cut-off criteria. This feature has the potential to be a true game-changer in treatment planning because until today the lack of knowledge of feasible dose sparing for a certain anatomy translated in a long sequence of manual trial-and-error attempts based on the skills of the planners. Since the choice of tradeoffs is planner dependent, manually generated plans may result in sub-optimal plans, potentially resulting in worse patient outcomes.

A major finding in this study is the impressive reduction of planning time provided by the Autoplanning and Personalized modules. For AP and Pers plans, the mean overall planning time, including human inputs, optimization loop and dose calculation, was determined to be around 23 and 10 min, respectively. However, it must be highlighted that the automated optimization of this challenging anatomical site required in all patients a postoptimization tuning. In this very challenging anatomical site, because of large tissue heterogeneity and irregular shape of the target volume, the optimization process could result in some dose discrepancy between dose objectives and finally calculated plans, often leading to locally high doses in the cord region or in the immediate vicinity. Even with the critical structures set to “none compromise,” the max dose to spinal cord could exceed the tolerance. In these cases, plans could been made clinically acceptable in a very easy way by performing a so-called warm restart, i.e., a manual fine tuning of the planning objectives based on the scorecard results. This final manual step does not require more than additional 5 min of dose calculation time.

We wish to emphasize that the use of a template-based optimization engine reduces all effort necessary for plan creation, beam setup, optimization objective definition, setup optimization and computation. In addition, a very large number of tuning optimization structures are automatically created during the loops optimization, which on the contrary would be especially time consuming if manually generated. Planning automation can overcome all criticism of this complex labor intensive manual procedure which prevents the use of SBRT for spine treatments in clinical routine, especially for patients requiring urgent management.

A main feature of this study is the implementation of a modified SIB strategy. The present treatment was focused on the BTV ablative dose escalation with dose fraction of 10 Gy, together with a lower safer dose fraction size to the entire vertebra (7 Gy/fraction), that should avoid failures at the epidural interface. In this way, the boost target received 50.0 Gy equivalent dose in 2 Gy/fraction (EQD2 Gy), whereas the surrounding vertebra received 42.5 Gy EQD2 Gy. We then aimed to achieve a significant increase in therapeutic gain as a result of the dual benefits of effective high tumor control and low treatment toxicity. In particular, we hypothesize that this strategy could decrease both the risk of marginal recurrences [seen in up to 12% of patients (35)] and vertebral collapse [currently estimated at 11%–20% (36)]. This approach has not yet been fully explored and only very few papers demonstrated that SIB strategy could be successfully applied to vertebral metastases in a dose-escalation trial (11–13).

It must be highlighted that spine SBRT planning using SIB technique involves a higher modulation of many machine parameters, resulting in more irregular beam apertures, larger tongue-and-groove effects, and larger dose-rate modulation, then placing higher demands on the accuracy of treatment machines and TPSs. This increasing complexity, in particular, may affect dose calculation uncertainties (due to limits in the algorithms or beam model) and the sensitivity of the delivered dose to small changes in machine parameters or patient geometry during treatment delivery (37). Therefore, patient-specific pretreatment verification must be considered mandatory to trace back any potential error in treatment planning process or machine deliverability. Regardless of plan complexity, there was an excellent agreement between measured and computed dose distributions for the 3% (global)/2 mm criterion (with 10% threshold and 90% passing rate) for all plans. These findings support the recommendations of the recent AAPM task group no. 218 (33) report on IMRT measurement-based verification quality assurance tolerance limits and procedures, confirming the deliverability of automated plans and their reliability and safety for clinical applications.Moreover, more complex plans usually necessitate longer beam-on durations, which can increase the risk of intrafraction motion (38). Because of their favorable characteristics such as the very high-dose rate and the low peripheral dose, all plans in this investigation were created for free-flattening filter (FFF) beams. The average delivery time of these spine treatments generated with FFF beams was found within 3.5 min, with an estimated reduction of about 8–10 min when compared with conventional FF beams treatment time delivery. This very short delivery time may potentially translate in safer treatments because of the expected reduction of intrafraction motion between setup and treatment completion.

Alternative algorithms for the automation of treatment planning have been implemented for spine SBRT (19–22). Compared with these alternative methods, the Autoplanning and Personalized engines present a clear advantage. Knowledge-based systems, for example, require a large library of prior high-quality plans to build up and train the corresponding mathematical model. Then, for each protocol and anatomical site, the clinical implementation translates in a labor intensive process. Each new generated plan hardly depends on the overall quality of previous plan used for modeling. Furthermore, any modifications to the contouring methodology, dose prescription, or planning techniques may need the creation of a new database. Template-based solutions, on the other hand, are unaffected by the quality of earlier plans, and template models can be generated without time consuming. In our experience, only three training patients were necessary as starting point for the implementation of the Technique by an expert team of medical physicists and radiation oncologists. With regard to the new Personalized module, it is worth noting that Feasibility calculates the fDVH for OARs from first principles, assuming simply that the targets are uniformly covered by the prescription doses and that no prior plan database is necessary.

A few limitations must be recognized. Firstly, we acknowledge that findings in our study are based on a small number of cases, which can impact statistical power estimations. Secondly, this dosimetric study investigated the feasibility of template-based automated planning for spine SBRT and the plan quality and delivery accuracy of automated generated plans. We did not address imaging requirements, patient setup reproducibility, and intrafraction treatment monitoring, which are all important aspects to ensure that the steep dose gradients are positioned correctly in relation to the target volume and spinal cord positions. Thirdly, it must be highlighted that the implementation and validation of a template-based model for clinical application requires a huge clinical experience of medical physicists, in order to wisely balance the tradeoffs between target coverage, dose conformity, and OAR sparing. Any suboptimal model implementation would results in systematic bias affecting all patients for that anatomical site. Lastly, it must be underlined that this is a single-institution study, i.e., local planning procedures may bias our findings which may not automatically transfer in other centers with different equipment, procedures or protocols.

Given the ablative doses and the potential for spinal cord injury, the current strategy should be implemented in clinical practice with special emphasis to patient setup immobilization and reproducibility. Nowadays, MRI-based image guidance has been implemented in clinical routine, and the first experiences with real-time MRI-guided radiotherapy for the delivery of SBRT for spinal metastasis have been recently reported (39). This novel technology, by increasing soft tissue contrast, can help to reduce the margin required for contouring uncertainty, organ motion, and intrafraction motion, hence limiting the underdosage of target epidural component where local failures are common.

In conclusion, Pinnacle3 Personalized plans outperformed conventional clinical plans in terms of dose conformity and dose contrast. Our results add to the growing literature new evidence that planning automation consistently generates high-quality plans with a major improvement of planning efficiency also for SBRT spinal treatments.

## Data Availability Statement

The raw data supporting the conclusions of this article will be made available by the authors, without undue reservation.

## Ethics Statement

The studies involving human participants were reviewed and approved by the Institutional Review Board of Gemelli Molise Hospital. The patients/participants provided their written informed consent to participate in this study.

## Author Contributions

SC conceived and designed the study. SC and FC drafted the manuscript. SC and CR planned the treatments and collected the data. FD, GM, MB, and LI analyzed the data and wrote the manuscript. PV performed patient-specific quality assurance. AM and VV made intellectual contribution and critical review of the manuscript. All authors revised the draft manuscript and approved the final version.

## Conflict of Interest

The authors declare that the research was conducted in the absence of any commercial or financial relationships that could be construed as a potential conflict of interest.

## Publisher’s Note

All claims expressed in this article are solely those of the authors and do not necessarily represent those of their affiliated organizations, or those of the publisher, the editors and the reviewers. Any product that may be evaluated in this article, or claim that may be made by its manufacturer, is not guaranteed or endorsed by the publisher.
